# Did the late spring frost in 2007 and 2011 affect tree-ring width and earlywood vessel size in Pedunculate oak (*Quercus robur*) in northern Poland?

**DOI:** 10.1007/s00484-015-1107-6

**Published:** 2015-11-25

**Authors:** Radosław Puchałka, Marcin Koprowski, Julia Przybylak, Rajmund Przybylak, Henryk P. Dąbrowski

**Affiliations:** 1 Herbarium TRN, Chair of Geobotany and Landscape Planning, Faculty of Biology and Environment Protection, Nicolaus Copernicus University, Lwowska 1, 87-100 Toruń, Poland; 2 Chair of Ecology and Biogeography, Faculty of Biology and Environment Protection, Nicolaus Copernicus University, Lwowska 1, 87-100 Toruń, Poland; 3 Nicolaus Copernicus University Academic Secondary School, Szosa Chełmińska 83, 87-100 Toruń, Poland; 4 Department of Meteorology and Climatology, Faculty of Earth Sciences, Nicolaus Copernicus University, Lwowska 1, 87-100 Toruń, Poland; 5 Archaeological Museum in Biskupin, Biskupin 17, 88-410 Gąsawa, Poland; 6 Medical University of Warsaw, ul. Żwirki i Wigury 61, 02-091 Warszawa, Poland

**Keywords:** Dendroecology, Dendroanatomy, Pedunculate oak, Late spring frost

## Abstract

Trees are sensitive to extreme weather and environmental conditions. This sensitivity is visible in tree-ring widths and cell structure. In our study, we hypothesized that the sudden frost noted at the beginning of May in both 2007 and 2011 affected cambial activity and, consequently, the number and size of vessels in the tree rings. It was decided to test this hypothesis after damage to leaves was observed. The applied response function model did not show any significant relationships between spring temperature and growth. However, this method uses average values for long periods and sometimes misses the short-term effects. This is why we decided to study each ring separately, comparing them with rings unaffected by the late frost. Our study showed that the short-term effect of sudden frost in late spring did not affect tree rings and selected cell parameters. The most likely reasons for this are (i) cambial activity producing the earlywood vessels before the occurrence of the observed leaf damage, (ii) the forest micro-climate protecting the trees from the harsh frost and (iii) the temperature decline being too short-lived an event to affect the oaks. On the other hand, the visible damage may be occasional and not affect cambium activity and tree vitality at all. We conclude that oak is well-adapted to this phenomenon.

**Electronic supplementary material:**

The online version of this article (doi:10.1007/s00484-015-1107-6) contains supplementary material, which is available to authorized users.

## Introduction

Cambial activity is largely modified by climate. In the temperate zone, the key factor controlling the cambium activation is temperature (Pukacka 2006), whereas in the tropical zone, this function is often taken on by precipitation (Wils et al. 2011). The influence of extreme weather phenomena is reflected in the formation of narrow or wide annual growth rings, which provide valuable environmental and bioindicative information. The width of annual rings and the density of the wood provide further insight into other environmental factors influencing growth, such as insect outbreaks. In addition to narrow or missing rings, wood density is also an indicator of insect outbreak (Koprowski and Duncker 2012). Anatomical characteristics, such as the size of the vessel lumen and their number, also have an indicative value (Campelo et al. 2010; Abrantes et al. 2012; Gricar et al. 2013). It is noteworthy that cellular parameters are not only used for dendroclimatological analysis but also serve dendrogeomorphological research (Koprowski et al. 2010; Wistuba et al. 2015), which considerably extends the application of dendrochronology.

**Fig. 1:**
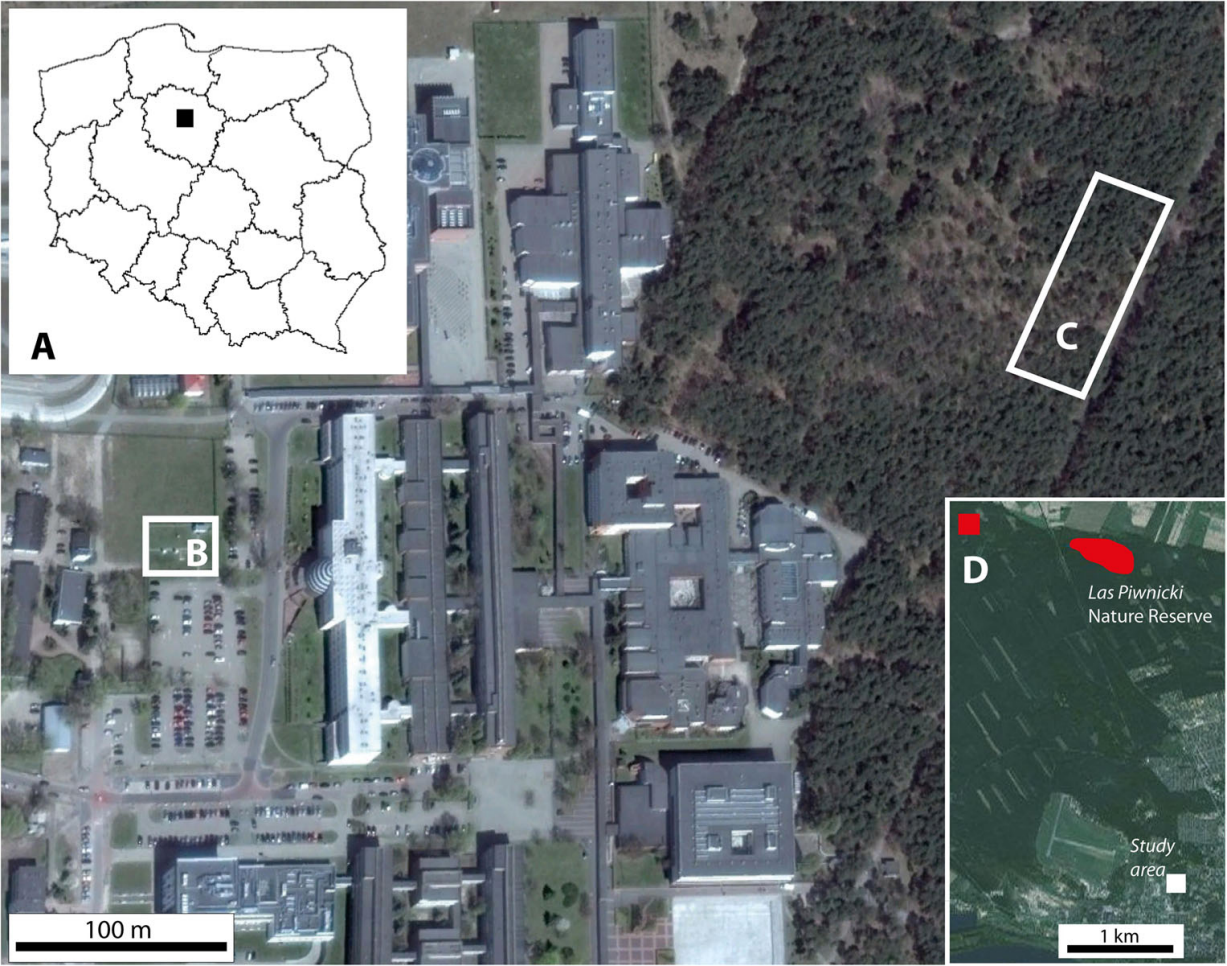
General location of the study area in Poland (**a**), meteorological station of the Department of Meteorology and Climatology, Nicolaus Copernicus University (**b**), research site in the forest near University campus (**c**) and reference sites marked in *red* (**d**)

**Fig. 2:**
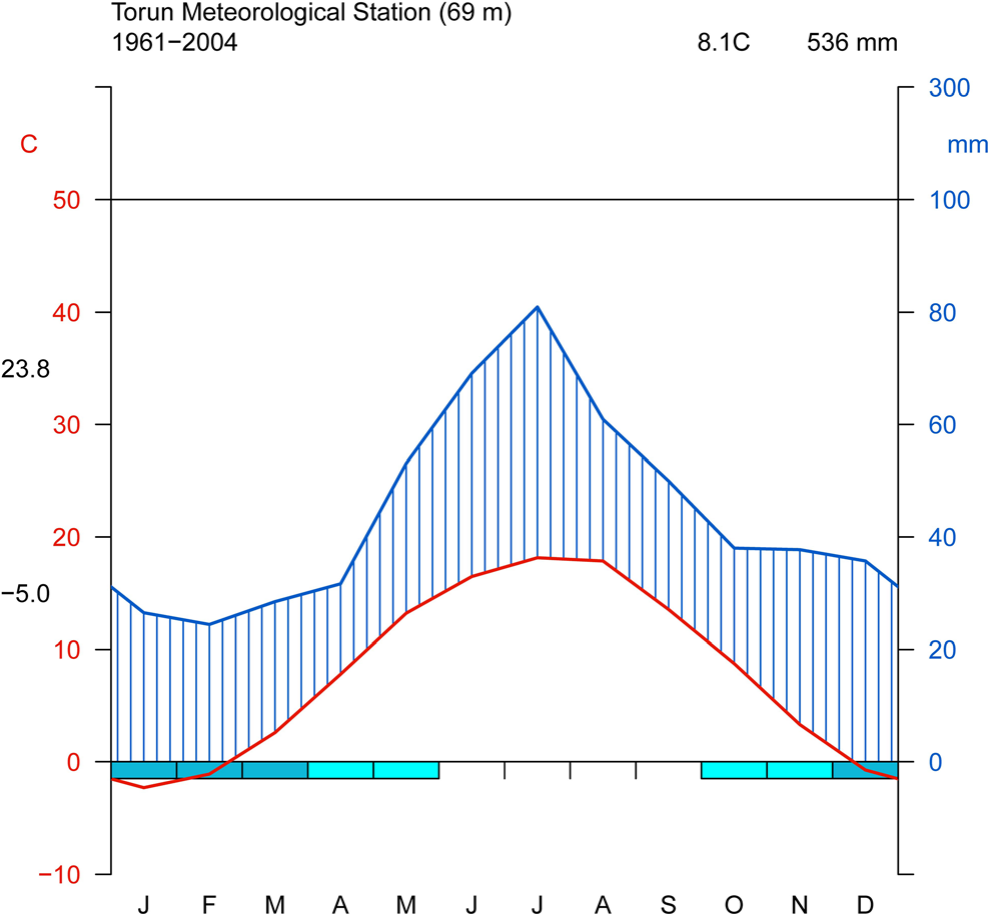
Climate diagram of the meteorological station in Toruń. Each tic mark along the abscissa indicates a month, *J* January, *F* February, etc. The diagram shows the appearance of daily minimum temperatures below zero in *blue bars* below the *horizontal line*. *Blue line* is a precipitation curve. *Red line*—temperature. 23.8 °C—mean daily max. temperature of the warmest month, −5.0 °C—mean daily min. temperature of the coldest month. *Upper right corner* of the diagram—annual average of temperature and annual precipitation sum (colour figure online)

**Fig. 3:**
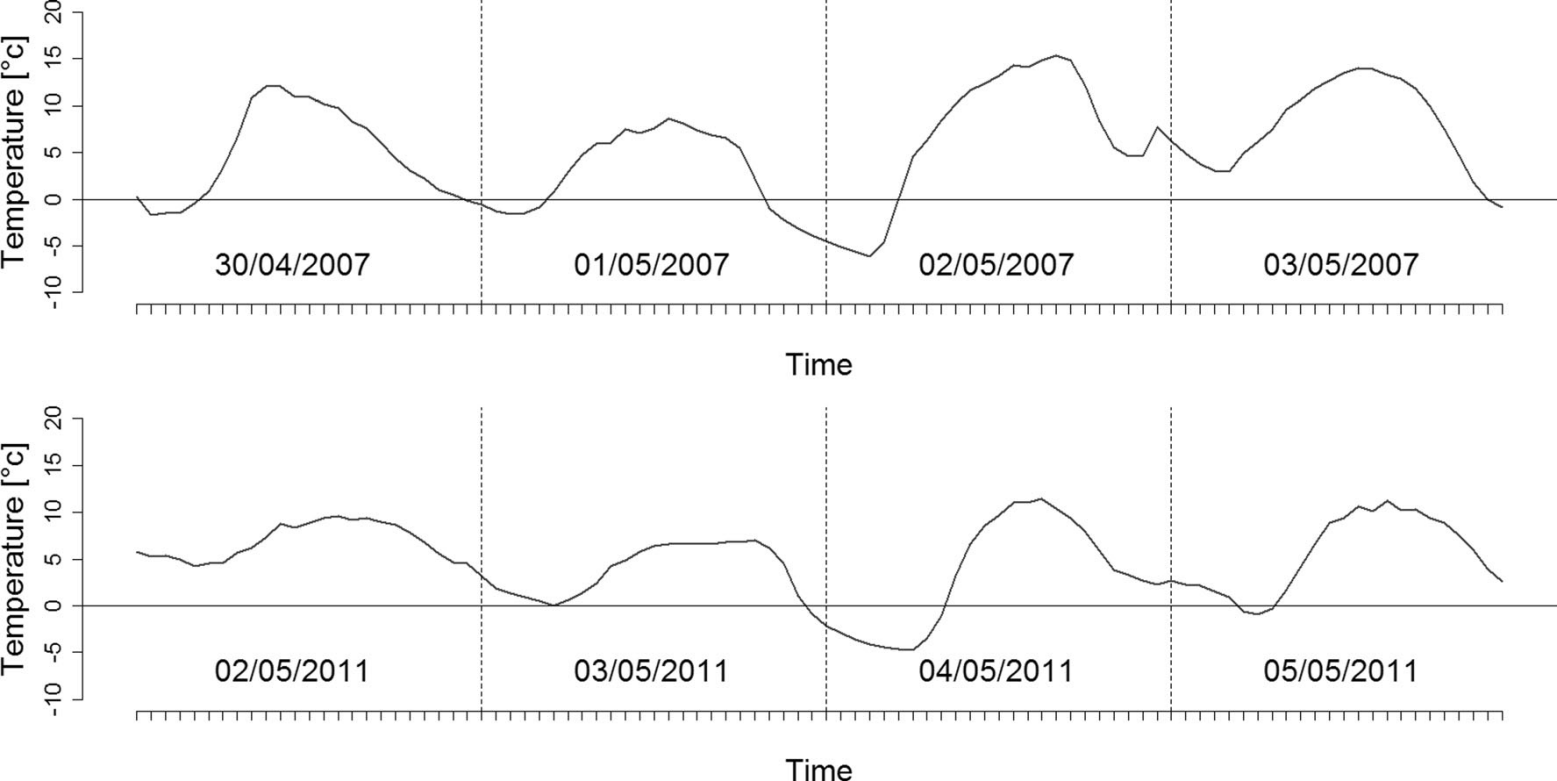
A decrease of temperature at the beginning of May in 2007 (from 30 April to 3 May) and 2011(from 2 May to 5 May)

**Fig. 4:**
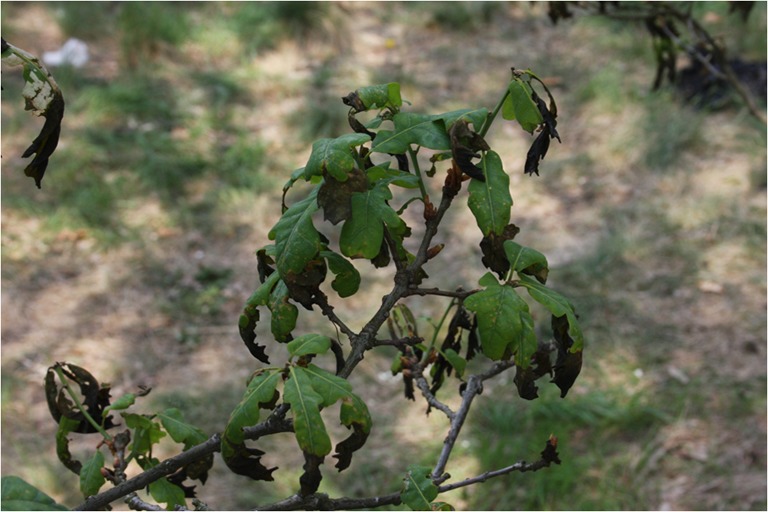
Damage of the oak leaves after the sudden frost on the 4th of May in 2011. Picture taken on the 11th of May

The research site is located near the University campus (Fig. 1). Ground frost occurs earlier and lasts longer (from 10 October to 5 May) in the vicinity of Toruń than in its surrounding areas (Woś 2010), and the meteorological spring begins quite late (5 May) (Fig. 2). At the end of April/beginning of May 2007 and 2011, frost occurred there early in the morning and persisted from 3 May until 6 May (Fig. 2). Springtime temperature drops could affect the differentiation of xylem cells in the period following the resumption of cambial activity (Hejnowicz 2002), and the sudden temperature decreases of 2007 and 2011 (Fig. 3) caused damage in oak leaves there (Fig. 4). The purpose of our study was to find out whether or not the late-spring ground frost, which causes damage to the assimilative apparatus, also affects tree-ring width and the number and size of vessels in the earlywood. In the case of ring-porous tree species, such as oak or chestnut trees, vessel size depends on the quantity of precipitation and the height of late-summer and early-spring temperatures that precede their development (Garcia-González and Eckstein 2003; Fonti et al. 2004; Fonti and García-González 2008; González-González et al. 2013b). On the other hand, no data is available on the damage to the assimilative apparatus or how it affects cambial activity. A hypothesis was that the damaged oak leaves had a negative effect on the activity of the cambium and on the anatomical characteristics of annual growth rings.

## Materials and methods

A total of 24 samples were taken from twelve oaks (two samples per tree) growing in the forest near the student campus of the Nicolaus Copernicus University in Toruń and the meteorological station of its Department of Meteorology and Climatology (Fig. 1). Samples were taken with a Pressler borer at breast height (130 cm above the ground). For reference, a local chronology was built for the oaks growing in the Toruń Forest District (Fig. 1). The aim in building the reference chronology was to study the long-term effect of climate on tree rings and to answer the question of whether it is dependent on temperature at the beginning of the vegetation season. The 24 samples from the 12 oaks were taken in 2003 from the ‘Las Piwnicki’ Nature Reserve, while 20 samples were taken from 10 trees in 2012 from a neighbouring site. The cores were prepared for measurement using standard dendrochronological procedures (Zielski and Krąpiec 2004). The sanded core samples were then scanned at a resolution of 1200 dpi using a standard scanner (Epson Perfection V700 Photo). Basic tree-ring parameters were obtained from the measurement of ring widths to the nearest 0.01 mm using CooRecorder software and the related CDendro programme (http://www.cybis.se). Checks on cross-matching were carried out using COFECHA (Grissino-Mayer 2001). In addition, each sample was analysed by means of the skeleton plot method (Schweingruber 1996). Both the skeleton plot method and the results from the COFECHA programme were used to evaluate and detect narrow and wide rings. Having checked the cross-matching, 28 samples were taken for study of climate-growth relationships. De-trending of the chronology was done with the dplR software (Bunn 2008) using the spline-smoothing option, which reflects trends in the chronology better than the other options. The ‘n-year spline’ was fixed at 2/3 the wavelength of the *n* years (Cook et al. 1990). A residual version of the chronology was built with pre-whitening, performed by fitting an autoregressive model to the data with AIC model selection (Bunn 2008).

To investigate climate/growth relationships for the reference chronology, the R package treeclim was applied (Zang and Biondi 2015) using a bootstrap procedure to estimate the degree of error. Climate data from October (previous year) to September (current year) served as independent variables, and the residual chronology for the site was used as dependent variables.

The trees examined in the earlywood vessel study were chosen randomly, were growing close together in a mixed pine-oak forest and were aged from 17 to 62 years. The level of damage between and within the trees (crown, lower and top branches) differed by phenophase (Fig. 1S). As discussed below (see Discussion), this was a tree-related factor. The cores (15–20 μm thick) were prepared using a Zeiss Hyrax S30 sledge microtome. Microscopic specimens were stained with safranin and mounted with a Heft Histokitt. Microscopic photographs were taken using a Moticam 580 camera and related software (Moticam Images Plus 2.0). The number and surface of earlywood xylem cells were measured with ImageJ (http://imagej.nih.gov/ij/), the Java-based image analysis programme, which showed a core width of 4 mm. The surface range of earlywood vessel cells considered was 0.005 to 0.25 mm^2^ (Garcia-González and Eckstein 2003). In order to determine whether the selected parameters were significantly different across individual years, a variance analysis (ANOVA) and a Tukey’s test were performed. Finally, the study covered tree rings from the years 2004–2012.

## Results

### Reference chronology and relationships between tree rings and climate

**Fig. 5:**
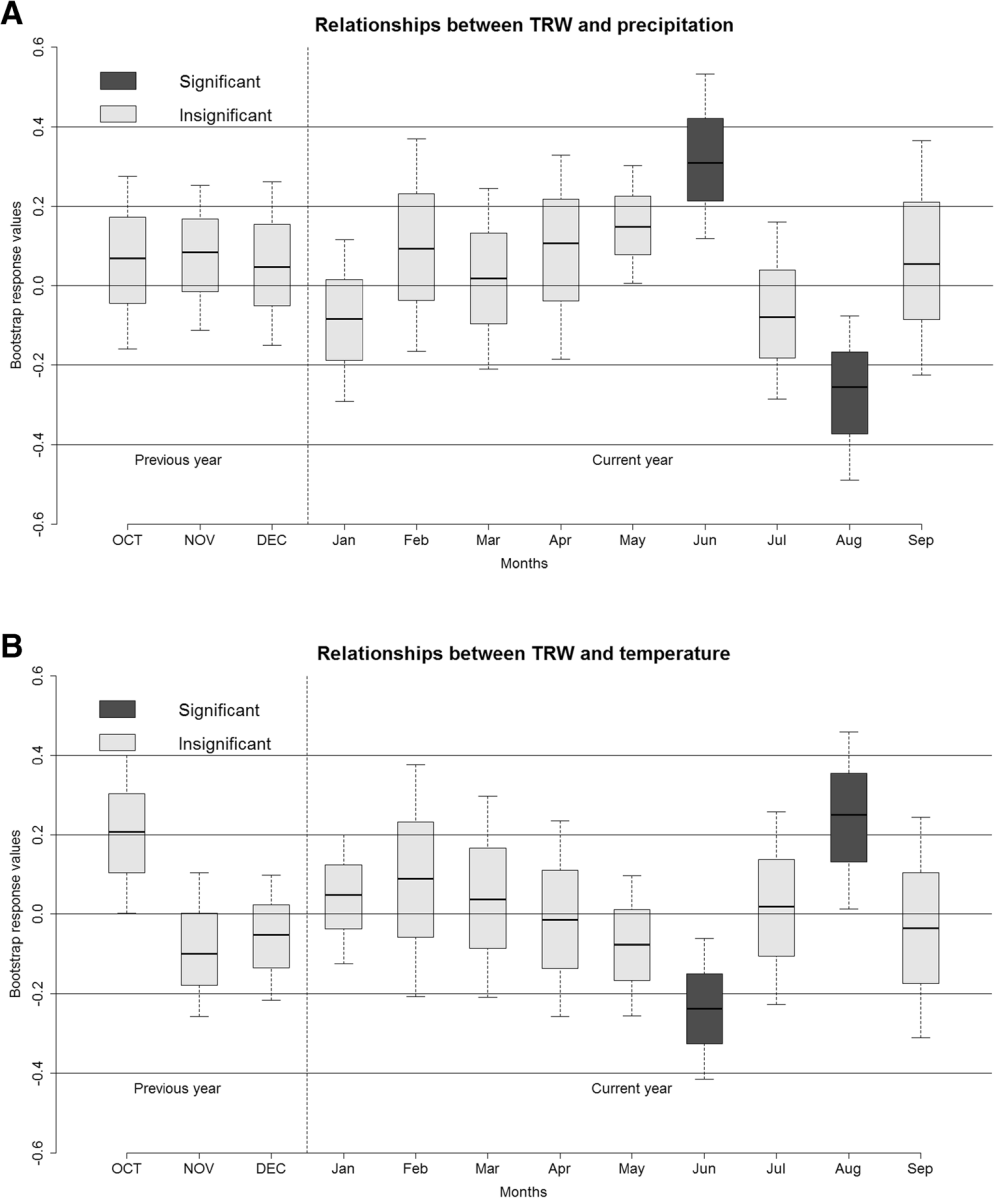
**a** Relationships between growth and precipitation. Significant values are at *p* < 0.05 in the certain months. **b** Relationships between growth and temperature. Significant values are at *p* < 0.05 in the certain months

The reference chronology covers the years 1714–2011, with a mean ring width of 1.56 mm. Standard deviation is 0.629, autocorrelation 0.755 and mean sensitivity 0.189. EPS value is 0.846 and is close to 0.85, usually treated as a value above which the climate response is strong. Significant (*p* < 0.05) positive relationships were observed in June precipitation and negative relationships in June temperature, while adverse significant (*p* < 0.05) relationships were noted in August (Fig 5a, b). The values of the bootstrap correlation between tree-ring chronologies, mean monthly temperatures and precipitation, were calculated for the period 1951–2011.

### Tree-ring width

**Fig. 6:**
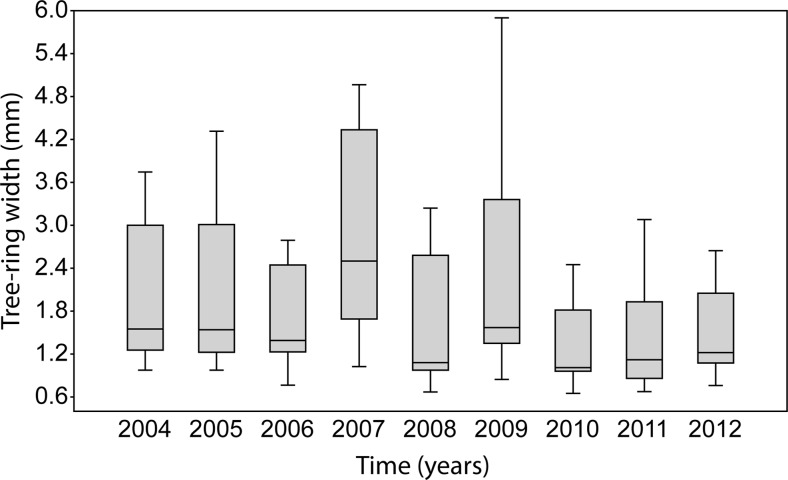
Box plot of tree-ring widths from 2004 to 2011

The average tree-ring width in the years 2004–2012 ranged from 1.278 mm in 2010 to 2.930 in 2007 (Fig. 6). Statistically, significant differences of *p* < 0.05 were observed between mean values of this parameter in subsequent years (Table 1), reaching its highest values in 2007. According to the Tukey’s test, the tree-ring width in 2007 was statistically different from the widths determined for 2006, 2008, 2010, 2011 and 2012. In the year 2011, when the late-spring frost occurred and damaged the leaves, this parameter was statistically different from the value observed in 2007 (Table 2).

**Table 1:** Results of ANOVA for mean values of tree-ring widths, earlywood vessel number and earlywood vessel area

Tree-ring characteristics		Sum of squares	*df*	Mean square	*F*	*p*
Tree-ring widths	Between groups:	27.1337	8	3.39171	**3.596**	**0.001053**
	Within groups:	93.3889	99	0.943322		
	Total:	120.523	107			
Earlywood vessel number	Between groups:	242.296	8	30.287	0.7928	0.6102
	Within groups:	3782.25	99	38.2045		
	Total:	4024.55	107			
Earlywood vessel area	Between groups:	1.19E + 08	8	1.48E + 07	0.1042	0.999
	Within groups:	1.41E + 10	99	1.42E + 08		
	Total:	1.42E + 10	107			

Statistically significant values are bolded

*df* degrees of freedom, *F F* test, *p* significance level

**Table 2:** Results of Tukey’s test values for tree-ring widths

	2012	2011	2010	2009	2008	2007	2006	2005	2004
2012		1	0.9999	0.3667	1	**0.01059**	1	0.9085	0.9387
2011	0.2229		1	0.2779	0.9999	**0.006333**	0.9997	0.8428	0.886
2010	0.6687	0.4458		0.1447	0.9968	**0.002188**	0.9931	0.6578	0.721
2009	3.219	3.442	3.888		0.5782	0.8889	0.6431	0.9918	0.9842
2008	0.4547	0.6777	1.123	2.764		**0.02844**	1	0.9807	0.9897
2007	5.222	5.445	5.891	2.003	4.767		**0.03747**	0.3356	0.2817
2006	0.59	0.8129	1.259	2.629	0.1352	4.632		0.9896	0.995
2005	1.929	2.152	2.598	1.29	1.474	3.293	1.339		1
2004	1.791	2.014	2.46	1.428	1.336	3.431	1.201	0.1382	

Bold values point out the significant differences between tree-ring widths in selected years

### Number of vessels

**Fig. 7:**
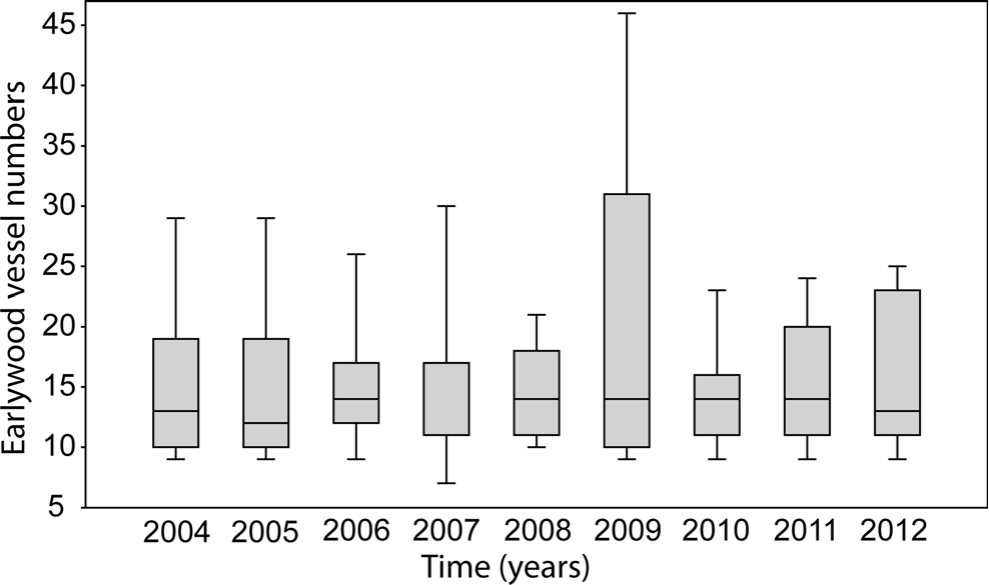
Box plot of earlywood vessel numbers from 2004 to 2011

The average number of earlywood vessels within the analysed tree-ring surface ranged from 14.250 in 2007 to 19.333 in 2009 (Fig. 7). In 2011, it reached 15.750. No statistically significant differences (*p* > 0.05) were identified for the tree rings from 2004 to 2012.

### Earlywood vessel area

**Fig. 8:**
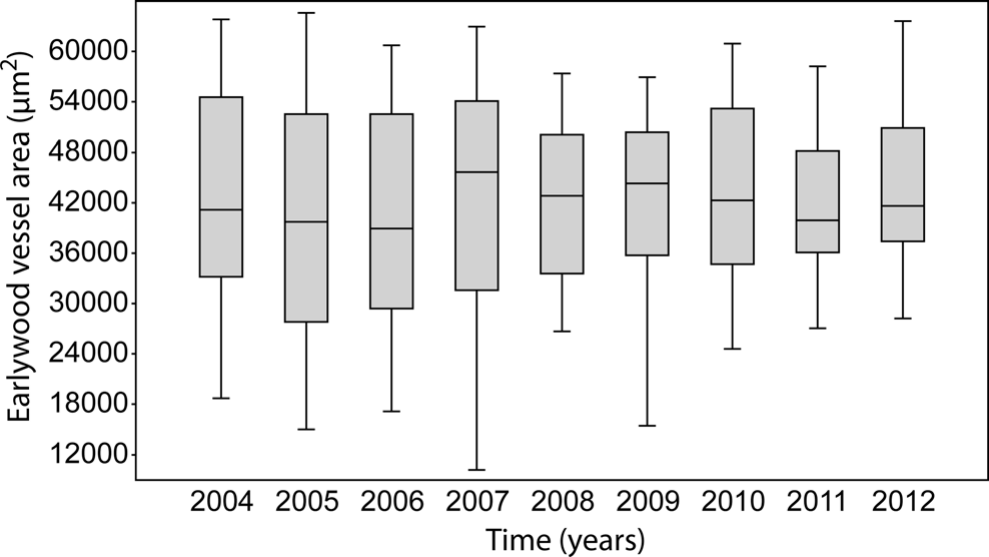
Box plot of earlywood vessel area from 2004 to 2011

The average cross-sectional area of earlywood vessels in 2004–2012 ranged from 40,155.02 μm^2^ in 2005 to 44,042.00 μm^2^ in 2012. In 2007, their size started at 43,027.81 μm^2^ and in 2011 at 42,096.5 μm^2^ (Fig. 8). No statistically significant differences (*p* > 0.05) were found in the vessel area in the years 2004–2012.

## Discussion

The origin of the question of the impact of sudden temperature drops on anatomical characteristics of annual growth rings was the damage observed to leaves in the first days of May and the research results indicating the influence of early-spring temperatures on the number and size of earlywood vessels (Garcia-González and Eckstein 2003; Fonti et al. 2004; Fonti and García-González 2004; García-González and Fonti 2006; Fonti and García-González 2008; González-González et al. 2013b). Our study did not validate the hypothesis that the springtime ground frost in early May 2007 and 2011 had a negative effect on either tree-ring width or the number and size of earlywood vessels (Figs. 6, 7 and 8; Tables 1 and 2).

Climatic requirements determined on the basis of trees growing in the area of Toruń show the substantial role of precipitation and temperature in June and August in a given year. Oaks in the area of Płońsk reveal a similar response (Bronisz et al. 2012). No influence of low temperatures in May on the anatomical characteristics in question was observed at any of the analysed sites. On the other hand, oaks declining in the south of Finland have proved sensitive to low May temperatures (Sohar et al. 2013). The oaks that we examined showed no pathological symptoms and thus were considered to be healthy. This may be the reason for their resistance to lower temperatures in May. Climatic conditions in the year preceding the growth affect tree-ring width in the given year. In ring-porous trees, such as oaks, chestnuts and ashes, the development of earlywood vessels begins some 2–3 weeks before the development of leaves (Vikhrov 1954; Ermich 1959; Zasada and Zahner 1971; Guzicka and Hejnowicz 2006; Pukacka 2006; Sass-Klaassen et al. 2011; González-González et al. 2013a). In this process, reserves accumulated in the previous growing season are used (Ermich 1959). The characteristics of a given annual growth ring can be very well predetermined by the number and size of vessels in the previous year’s ring. This is due to the frequent closure of earlywood vessel lumen by tyloses, which deprive the vessels of their transport capacity and affect its efficiency in the subsequent growing season (Ermich 1959; Guzicka and Hejnowicz 2006).

As much as 75 % of water is conducted through the youngest growth ring, whereas the remaining 25 % is transported by the four outermost rings (Guzicka and Hejnowicz 2006).

Trees growing in the north of Poland revealed a distinct correlation between tree-ring width and the amount of summer precipitation in the preceding year (Ważny 1990). The greater role of pluvial—rather than thermal—conditions, may indicate the lack of a clear response in tree-rings to the May frost in the analysed oaks. However, other authors have underlined the role of temperature in the development of tree-ring width (Karolewski 2006). Such ambiguity may be due to the origin of a given tree population or interactions between temperature, soil moisture and air humidity (Karolewski 2006).

In the case analysed, ground frost appeared when the leaves were in a different stage of development (on the 124th day of the year). In some individuals, the buds were swollen, and in other trees, the leaves were nearly fully expanded. Our observations point to the studied trees representing different times of phenophases, and this can be related to the co-occurrence of two ecotypes: *Q. robur* fo. *praecox* (early oak), and *Q. robur* fo. *tardifolia* (late oak) (Vikhrov 1954; Wesołowski and Rowiński 2008). In ring-porous trees, no explicit correlations between the formation of earlywood vessel cells and leaf phenology were found (Sass-Klaassen et al. 2011). The development of earlywood vessels begins later than would follow from earlier research (Sass-Klaassen et al. 2011). In oaks, the vessels start to expand when the buds are already swollen (Zasada and Zahner 1971; González-González et al. 2013a). By the time leaf development is completed, approximately 80 % of earlywood vessels have already developed, unlike in ash trees where the development of vessels begins and ends earlier (Sass-Klaassen et al. 2011). Taking into account the study by Sass-Klassen et al. (2011), which shows that differences in time xylogenesis and leaf phenology between individual trees exist, and considering our own observations of the studied population (Puchałka et al. (in preparation), Puchałka et al. 2015), we can assume that earlywood vessel formation was incomplete before the leaves had fully expanded. Therefore, the 2011 ground frost occurred when earlywood vessels were in the development stage and the stress from the damage of the assimilative apparatus had been compensated for by the favourable weather conditions in the later part of the growing season. Exact specification of the time of the earlywood vessel cells’ formation may be difficult, due to the diverse weather conditions in different calendar years. In Turkey, for example, the cambial activity of sessile oaks began in 2003 in the first week of May, whereas in the following year, it began a month earlier (Akkemik et al. 2006). Furthermore, the Pedunculate oak is quite a variable species in terms of phenology. Within its population, differences in leaf formation ranging from a few days to 5 weeks have been observed in various areas of its geographical range (Vikhrov 1954; Ermich 1959; Wesołowski and Rowiński 2008; Sass-Klaassen et al. 2011; González-González et al. 2013a). Some authors claim that phenological variability within this species proves its adaptation to extreme weather conditions and insect outbreaks (Vikhrov 1954; Wesołowski and Rowiński 2008). Of the different parts of the geographical range of the Pedunculate oak, the sympatric existence of two forms of this species—an early *Q. robur* fo. *praecox* and a late *Q. robur* fo. *tardifolia*—has been observed (Vikhrov 1954; Kleinschmit 1993; Rubtsov 1996; Wesołowski and Rowiński 2008; Batos et al. 2012; Bobinac et al. 2012). It should also be noted here that phenological phases can follow diverse patterns in subsequent growing seasons (Ermich 1959). Differences between individual specimens may be due to ontogenetic factors, accumulated carbohydrate quantities (Pukacka 2006), hormone concentrations (Michalski and Krzyśko 1970) and genetic variation (Ueno et al. 2011). As far as environmental factors are concerned, the competition for water and light can be decisive. This is manifested in the extent of the tree’s root system and the size and shape of its crown (Akkemik et al. 2006). Comparative studies of the closely-related *Q. petraea* and *Q. pyrenaica* species have demonstrated that the first of these two—being also very diverse in terms of phenology—shows a much less evident response to extreme weather conditions (González-González et al. 2013b). *Quercus petraea* and *Q. robur* are the most wide-spread oak species in Europe, growing in considerably diverse habitats and climates (Boratyński et al. 2006; Danielewicz and Pawlaczyk 2006). This indicates that phenological variability may be an alternative response in xylem structure and a proven form of adaptation to environmental conditions. A more detailed explanation of the response to extreme weather conditions requires further research, considering the physiological state of trees, the genetic variability of a tree population and the role of individual specimens in a community.

## Conclusions

The results of our studies did not prove the hypothesis of the influence of the sudden ground frost in May on the anatomical characteristics of the annual growth ring. The influence of the ground frost in 2007 was compensated for by high temperatures in May, July and August, which could affect the greater width of tree rings in comparison with previous years’ values. The average temperature in May 2011 (14.5 °C) was also 0.8 °C higher than the long-term mean. Temperature conditions which favour tree growth could compensate for any negative influence of ground frost. The possibility that the ground frost events in Toruń in May 2011 were too insignificant and short-lived to cause any substantial disturbance (e.g. to reduce the number of earlywood vessels) in the tree rings of the growing oaks also cannot be ruled out. In spite of having sourced climate data from a meteorological station situated at a distance of approx. 0.5 km from the studied tree stands, the data does not fully represent the thermal conditions prevailing in the forest. Normally, forest areas are warmer at night and at dawn than open areas.

The results obtained have inspired us to undertake further studies into cambial activity in the growing season and to describe it using diurnal weather extremes and leaf phenology.

## Electronic supplementary material

Fig. 1S Different level of leaves damage dependably on the position within individual tree. (JPEG 1191 kb)
