# Supplementary figures and images for: Did the late spring frost in 2007 and 2011 affect tree-ring width and earlywood vessel size in Pedunculate oak (Quercus robur) in northern Poland?
Source: Int J Biometeorol. 2015 Nov 25;60:1143–50. doi: 10.1007/s00484-015-1107-6 (PMC4961729; doi:10.1007/s00484-015-1107-6)

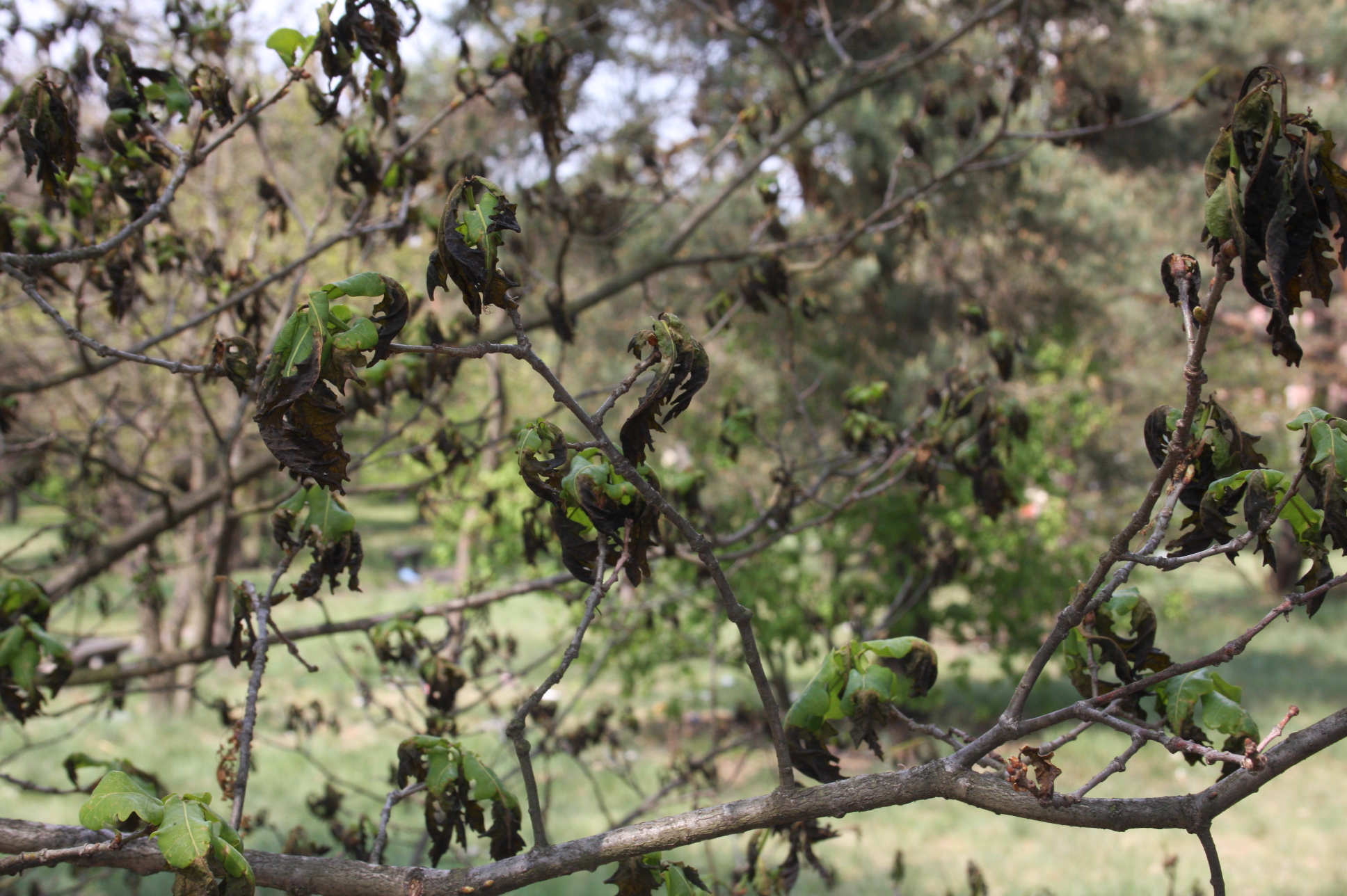

Supplement: Supplementary file 1 — Different level of leaves damage dependably on the position within individual tree. (JPEG 1191 kb) [file 484_2015_1107_Fig9_ESM.jpg]
